# Identification of novel alternative splicing biomarkers for breast cancer with LC/MS/MS and RNA-Seq

**DOI:** 10.1186/s12859-020-03824-8

**Published:** 2020-12-03

**Authors:** Fan Zhang, Chris K. Deng, Mu Wang, Bin Deng, Robert Barber, Gang Huang

**Affiliations:** 1 Vermont Biomedical Research Network and Department of Biology, University of Vermont, Burlington, VT 05405 USA; 2 Institute for Translational Research and Department of Family Medicine, University of North Texas Health Science Center, Fort Worth, TX 76107 USA; 3 School of Molecular and Cellular Biology, University of Illinois at Urbana–Champaign, Champaign, IL 61801 USA; 4 Department of Biochemistry and Molecular Biology, IU School of Medicine, Indianapolis, IN 46202 USA; 5 Indiana Center for Systems Biology and Personalized Medicine, Indianapolis, IN 46202 USA; 6 Department of Pharmacology and Neuroscience, University of North Texas Health Science Center, Fort Worth, TX USA; 7 Shanghai Key Laboratory for Molecular Imaging, Shanghai University of Medicine and Health Sciences, Shanghai, 201318 People’s Republic of China

**Keywords:** Alternative splicing, Breast cancer, Biomarker discovery, Pathway analysis, Mass spectrometry

## Abstract

**Background:**

Alternative splicing isoforms have been reported as a new and robust class of diagnostic biomarkers. Over 95% of human genes are estimated to be alternatively spliced as a powerful means of producing functionally diverse proteins from a single gene. The emergence of next-generation sequencing technologies, especially RNA-seq, provides novel insights into large-scale detection and analysis of alternative splicing at the transcriptional level. Advances in Proteomic Technologies such as liquid chromatography coupled tandem mass spectrometry (LC–MS/MS), have shown tremendous power for the parallel characterization of large amount of proteins in biological samples. Although poor correspondence has been generally found from previous qualitative comparative analysis between proteomics and microarray data, significantly higher degrees of correlation have been observed at the level of exon. Combining protein and RNA data by searching LC–MS/MS data against a customized protein database from RNA-Seq may produce a subset of alternatively spliced protein isoform candidates that have higher confidence.

**Results:**

We developed a bioinformatics workflow to discover alternative splicing biomarkers from LC–MS/MS using RNA-Seq. First, we retrieved high confident, novel alternative splicing biomarkers from the breast cancer RNA-Seq database. Then, we translated these sequences into in silico* Isoform Junction Peptides*, and created a customized alternative splicing database for MS searching. Lastly, we ran the Open Mass spectrometry Search Algorithm against the customized alternative splicing database with breast cancer plasma proteome. Twenty six alternative splicing biomarker peptides with one single intron event and one exon skipping event were identified. Further interpretation of biological pathways with our Integrated Pathway Analysis Database showed that these 26 peptides are associated with Cancer, Signaling, Metabolism, Regulation, Immune System and Hemostasis pathways, which are consistent with the 256 alternative splicing biomarkers from the RNA-Seq.

**Conclusions:**

This paper presents a bioinformatics workflow for using RNA-seq data to discover novel alternative splicing biomarkers from the breast cancer proteome. As a complement to synthetic alternative splicing database technique for alternative splicing identification, this method combines the advantages of two platforms: mass spectrometry and next generation sequencing and can help identify potentially highly sample-specific alternative splicing isoform biomarkers at early-stage of cancer.

## Background

Breast cancer death rates for women in the U.S. are higher than those for any other cancer, besides lung cancer. About 1 in 8 U.S. women (about 12%) will develop invasive breast cancer over the course of her lifetime. In 2020, an estimated 276,480 new cases of invasive breast cancer were expected to be diagnosed in women in the U.S., along with 48,530 new cases of non-invasive (in situ) breast cancer [1].

Traditional methods for early breast cancer detection such as self-examination and mammography have many drawbacks. For example, tissue biopsies can be difficult to be obtained and small tumors may not be detected by mammography [2]. In recent years, alternative splicing variants (AS) have been reported to show their growing importance in representing a new class of diagnostic biomarkers [3–5]. Alternative splicing occurs during the splicing process of pre-mRNA in which introns are removed and exons are connected. Recent studies of genome-wide alternative splicing analysis estimated that over 95% of human genes contain alternative splicing events [6]. Alternative splicing have been reported to be implicated in several areas of cancer genesis and progression. For example, Yae et al. found that the expression of a CD44 variant isoform (CD44v) was regulated by epithelial splicing regulatory protein 1, and CD44 isoform could be switched from CD44v to CD44 standard (CD44s) by knockdown of epithelial splicing regulatory protein 1 in CD44v+ cells. Therefore, regulation of isoform CD44v was a potential therapeutic target to prevent metastasis [7].

Meanwhile, various platforms and methods have been designed for the purpose of identification of alterative splicing events, such as the Affymetrix Exon–Exon Junction Array, RNA-Seq, and LC–MS/MS. For example, Lapuk et al. used the Affymetrix Human Exon–Exon Junction Array to assess the level of alternative splicing in 31 breast cancer and nonmalignant immortalized cell lines which represented luminal, basal, and claudin-low breast cancer subtypes. 181 splice events were identified representing 156 genes as candidates for AS and 90% of them were confirmed as the predicted AS events by reverse transcription-PCR. Nearly half of the AS events were found to be associated with basal, luminal, or claudin-low breast cancer subtypes [8]. The Affymetrix Human Exon–Exon Junction Array they used has a significant improvement in probe design compared to the Affymetrix’s first generation exon array platform. The Affymetrix Human Exon–Exon Junction Array has on average eight probes per probeset, contains 315,137 exons and 260,488 exon–exon junctions, and covers all AS events in the UCSC/Ensembl databases [9].

The recent RNA-Seq technology has greatly revolutionized the way for alternative transcripts identification and quantification [10]. For example, with RNA-Seq, Eswaran et al. systematically revealed splicing characteristics of the three breast subtypes: TNBC, non-TNBC and HER2-positive and discovered subtype-specific differentially spliced genes and splice isoforms which were not previously recognized in human transcriptome. They validated novel isoforms of critical genes like CDK4, LARP1, ADD3, and PHLPP2. They found the predominant splice events in breast cancer: exon skipping and intron retention [11]. Unlike the Affymetrix Human Exon–Exon Junction Array, which can only measure pre-specified variants, RNA-Seq allows for the detection of novel splice junctions and exonic sequences [12]. A comparison of RNA-Seq and Affymetrix Human Exon–Exon Junction Array showed that RNA-Seq had significantly improved transcript coverage and increased sensitivity for differentially expressed transcripts [13].

It becomes possible to perform high-throughput AS analysis with recent advances in methodology, including expressed sequence tagged (EST) sequencing, exon array, exon–exon junction array, and next-generation sequencing of all mRNA transcripts [11]. However, “indirect” transcriptome-level characterization of alternative splicing has some disadvantages. For example, proteins are the actual major molecules in the cell but don’t often correlate well with mRNA transcripts.

Recently, as an innovative analytical technology platform, liquid chromatography tandem mass spectrometry (LC–MS/MS) has emerged and been applied to a wide number of analyses including high-throughput protein identification [14]. Both sensitivity and specificity of candidate disease biomarkers can be improved when using LC–MS/MS to identify alternative splicing isoforms relevant to disease. Many proteins can produce abundant pathological alternative splicing isoforms. It is often sufficient to identify them distinguishing between disease samples and controls because they may be exclusively regulated in a disease condition [15].

Searching against a proper alternative splicing database, tandem mass spectrometry can be powerful technique to identify, analyze and characterize potential novel alternative splicing isoforms at the protein level. For example, the synthetic AS databases PEPPI [15] and SASD [16] have demonstrated as efficient means for identification, analysis and characterization of novel AS isoforms from tandem mass spectrometry. The SASD [16] is superior to PEPPI [15] in that it supports context-specific alternative splicing searching, which enables users to focus on the specific proteins of interest and therefore remarkably reduce computational time. In addition, SASD also allows interpretation and analysis of alterative splicing events in the context of: pathway, disease, drug and tissue specificity.

A sample-specific protein database from RNA-Seq data should provide a more accurate profile of the real protein pool in the sample. The genome is relatively static, but the proteome varies with disease state, tissue nature, cell development stage, and effects of drug treatment. The interest in proteomics for the sequencing of the human genome has increased because DNA sequence provides a static snapshot in which the cell might use its proteins and holds great promises of proteomic dynamic process in the cell. Therefore, combining the two platforms by searching LC/MS/MS data against a sample-specific protein database from RNA-Seq may yield with a higher confidence a subset of alternatively spliced protein isoform candidates, although poor correlation between the two has been generally found [17–20].

Liquid chromatography tandem mass spectrometry (LC/MS/MS) proteomics analysis in combination with RNA-Seq has opened up new opportunities in biomarker discovery. For example, Wang et al. analyzed matched RNA-Seq and LC–MS/MS proteomics data and found that customized protein sequence databases significantly increased the sensitivity of peptide identification, reduced ambiguity in protein assembly, and enabled the detection of known and novel peptide variants [21]. They also proposed a workflow for constructing sample-specific protein sequence databases from RNA-Seq data for more sensitive and sequence variant-inclusive proteomic studies. Sheynkman et al. [22] collected RNA-Seq data and proteomics data from same cell population, and developed a bioinformatics pipeline to build customized databases for the discovery of novel splice-junction peptides.

We have developed a bioinformatic workflow to identify alternative splicing biomarkers from proteome using RNA-Seq. Using the RNA-Seq data from 8 normal and 24 breast tumor samples and the LC–MS/MS data from 40 normal and 40 breast cancer plasma samples, we demonstrated that the bioinformatic workflow can help identify novel alternative splicing biomarkers from proteome using RNA-Seq and is a complement to our SASD searching method.

## Results

**Fig. 1:**
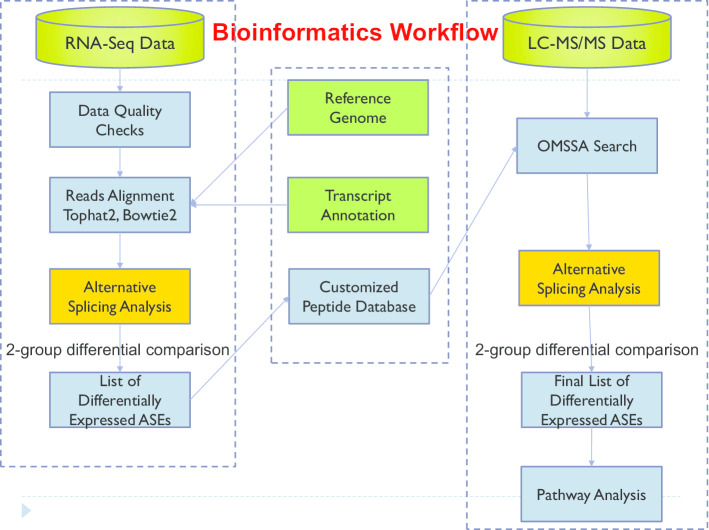
A bioinformatic workflow for identifying alternative splicing biomarkers from proteome using RNA-Seq

We developed a bioinformatics workflow (Fig. 1) for identifying alternative splicing biomarkers from the proteome using RNA-Seq, which contains three steps as described in detail in the “Methods and materials” section. In order to demonstrate that the bioinformatics workflow can identify alternative splicing biomarkers from the proteome using RNA-Seq, we collected breast cancer datasets from two platforms: LC–MS/MS and RNA-Seq.

**Fig. 2:**
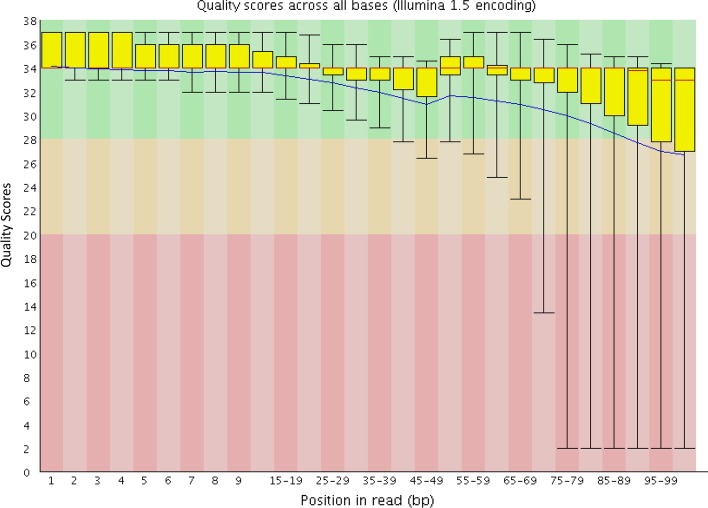
Per base sequence quality

The plasma proteome data were collected in the triple-play mode from 40 healthy women and 40 women diagnosed with breast cancer [23, 24]. The RNA-Seq fastq files were downloaded from the NCBI GEO database, which contained 8 normal samples and 24 breast cancer samples. The average number of RNA-Seq Reads in the fastq files was 29,012,741 (Table 1). We ran the FastQC to check the quality of the fastq files. The quality of the RNA-Seq data is uniformly good. Most the 32 fastq files had Per Base Sequence Quality scores all above 28 (Fig. 2). Some of the 32 fastq files were good up to about 90 bp and dropped right at the end. After trimming all the reads back to about 90 bp to remove the poor quality sequence, the RNA-Seq data were qualified for downstream analysis.

**Table 1:** Bioinformatic workflow numbers

	Count
Average number of RNA-Seq reads per sample	29,012,741
Junction with 6+ reads	186,784
Annotated junctions	175,225
Alternative splicing biomarkers from RNA-Seq (0.05 q-value)	256
Peptides 1% FDR and 0.1 E-value	79
Alternative splicing peptides from proteome	26

### Identification of alternative splicing biomarkers from RNA-Seq

#### RNA-Seq splice junction analysis

**Fig. 3:**
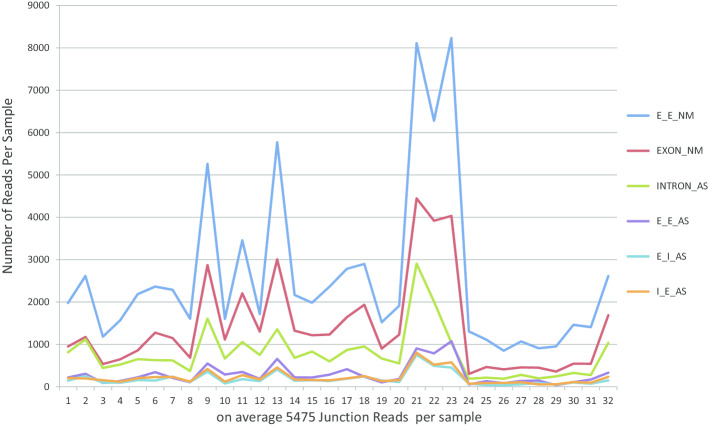
Junction read from RNA-Seq

**Fig. 4:**
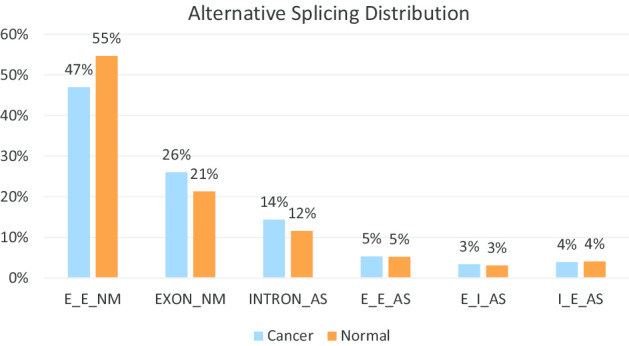
Alternative splicing distribution at normal and cancer states

First, we mapped the 186,784 junction reads into the transcript database in our SASD. And then, we calculated the overlap rates between each block of junctions and each exon or intron within the corresponding transcript. Lastly, we annotated the junctions by assigning the block in junctions to the exon or intron with a minimum overlap rate of 90% or above. After removing all unannotated junctions, we obtained 175,225 alternative splicing junctions, the majority of AS junctions were neighboring exon–exon junction (E_E_NM) and first category events (EXON_NM and INTRON_AS) (Fig. 3). We also observed that the distribution of alternative splicing junctions were similar at normal and cancer states, whereas the majority of alternative splicing junctions were neighboring exon–exon junction (E_E_NM) and first category events (EXON_NM and INTRON_AS) (Fig. 4).

We divided the 32 RNA-Seq samples into two groups: normal group (8 benign breast lesions) and breast cancer group (8 ER+, 8 triple negative, and 8 HER2+ primary breast tumors). With the statistics method described in the “Methods and materials” section, 256 alternative splicing biomarkers from 65 genes and 140 transcripts were found to be differentially present (q-value < 0.05) depending upon diagnostic status. Of these, 68 were single exon, 140 neighboring exon–exon junction, 5 exon skipping, 40 single intron retention, 2 left intron retention, and 1 right intron retention (Table 2). Further pathway analysis showed that the 65 genes were associated with Signaling, Cancer, Translation, Metabolism, Immune System and Hemostasis, which are consistent with previous findings.

**Table 2:** statistics of 256 alternative splicing biomarkers identified from RNA-Seq

Alternative splicing events	Count
EXON_NM	68
E_E_NM	140
E_I_AS	1
I_E_AS	2
E_E_AS	5
INTRON_AS	40
Total	256
Genes	65
Transcripts	140

### Construction of customized alternative splicing biomarker peptide database

We used the pipeline we developed for SASD [16] to convert the 256 alternative splicing biomarker sequences into putative polypeptide entries for mass spectrometry searching. Three types of common splicing events were contained in the customized alternative splicing peptide database. They are Normal Splicing [single exon (EXON_NM) and neighboring exon-–exon junction (E_E_NM)], Exon Skipping [non-neighboring exon–exon junction (E_E_AS)], and Intron Retention [single intron (INTRON_AS), left intron retention junction (I_E_AS), right intron retention junction (E_I_AS)].

### Identification of alternative splicing biomarkers from proteome

The OMSSA search yielded 79 peptides passing 1% MS/MS FDR and 0.1 E-value. With a significance (q < 0.05) and at least two hits from either cancer group or normal group as thresholds, we identified 26 alternative splicing biomarkers (Table 3), of which 1 is exon skipping (E_E_AS), 1 single intron retention (INTRON_AS), 21 single exon (EXON_NM), and 3 neighboring exon–exon junctions (E_E_NM). The three-step q-value method was described in detail in the “Methods and materials” section. The feature importance was determined by comparing the size of these coefficients to each other using five-fold cross-validation SVM model.

**Table 3:**
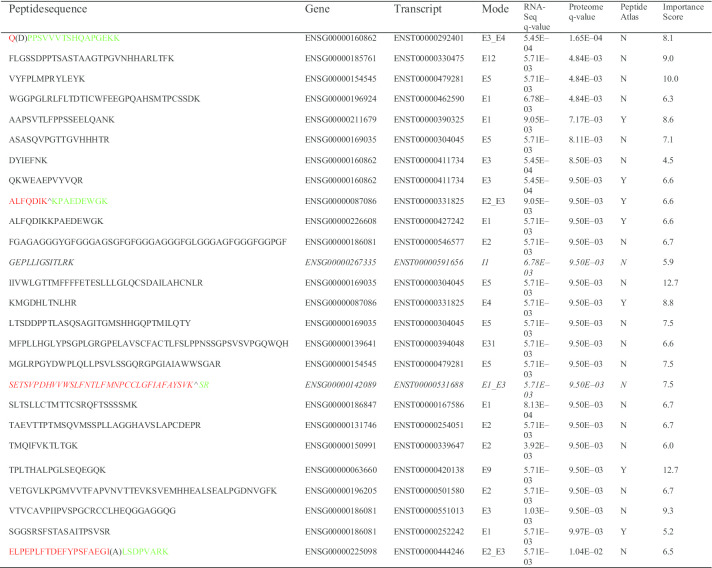
26 cancer-specific peptide markers identified in breast cancer plasma proteome

**Fig. 5:**
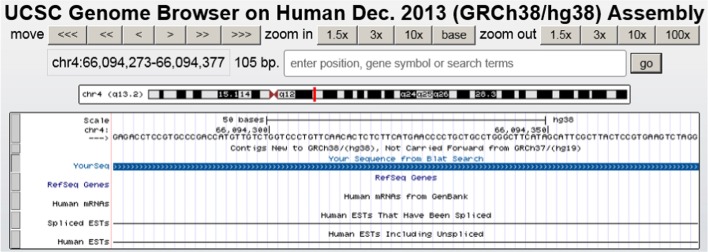
UCSC genome browser screen shot of genomic region for the novel peptide

Red color is the left part of the junction and green color is the right part. Splicing site is marked by ^ or ().‘^’ means the splicing site is separated by the left region and right region. ‘()’ means the splicing site is shared by both left and right regions. For example, the exon-skipping peptide  is a synthetic product of the ENST00000531688 in gene ENSG00000142089 (*IFITM3*) where the second exon was skipped and the first exon was combined together with the third exon. A screen shot from the UCSC genome browser [25] in the region of the peptide shows that the peptide sequence was not reported in EST sequences and mRNA from Genbank and refseq gene (Fig. 5).

Similar UCSC genome browser analysis for other peptides confirmed that 13 of the 26 peptides were not found in EST sequences and mRNA from Genbank and refseq genes. We also searched the Peptide Atlas database and found that 19 of the 26 peptides were not reported in the Peptide Atlas database (Table 3).

**Fig. 6:**
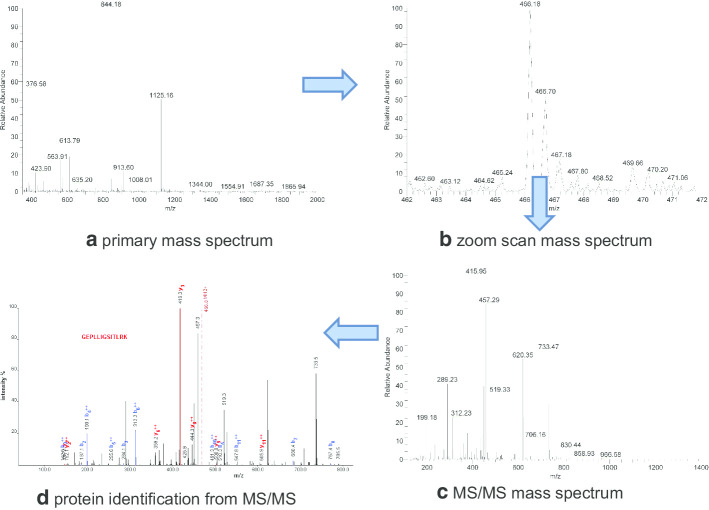
Triple play mode spectrum of GEPLLIGSITLRK. The blue lines represent b-ions. And the red lines represent y-ions

The single intron alternative splicing GEPLLIGSITLRK was identified in 10 cancer samples and 1 health sample. The triple play mode shown in the Fig. 6 includes (a) primary scan; (b) zoom scan; (c) MS/MS scan and (d) protein identification from MS/MS). The spectrums of other AS sequences were omitted due to space limit.

Our bioinformatic workflow has shown significant potential to discover new classes of high-quality alternative splicing biomarkers from mass spectrometry using RNA-Seq (Table 3). We also examined their prediction performance by modeling a Support Vector Machine with fivefold cross-validation and obtained a high performance (AUC = 0.9205, precision = 83.7%, accuracy = 86.3%, sensitivity = 90.0%, specificity = 82.5%, NPP = 89.2%) on the 80 breast cancer proteomic samples. A literature search found no any reports for the 26 peptides. Pathway analysis with the IPAD [26] shows the 26 peptides are associated with Cancer, Signaling, Metabolism, Regulation, Immune System and Hemostasis pathways, which was consistent with the 256 alternative splicing biomarkers from RNA-Seq.

## Discussion

We describe here a bioinformatic workflow to identify alternative splicing biomarkers from proteome using sample-specific RNA-Seq database. With the bioinformatics workflow, we identified 256 alternative splicing biomarkers from 65 genes and 140 differentially present transcripts (q-value < 0.05) at cancer state, out of which there are 68 single exon, 140 neighboring exon–exon junction, 5 exon skipping, 40 single intron retention, 2 left intron retention, and 1 right intron retention. And then, we built customized sample-specific peptide database of the 256 alternative splicing using the method we developed for our SASD [16]. Lastly, we discovered 26 alternative splicing biomarkers from proteome, of which 1 is exon skipping (E_E_AS), 1 single intron retention (INTRON_AS), 21 single exon (EXON_NM), and 3 neighboring exon–exon junctions (E_E_NM).

The number of alternative splicing biomarkers dropped dramatically from 256 in the peptide database to 26 in the proteome. The low number of alternative splicing biomarkers actually identified at the protein level is the major issue of the bioinformatics workflow. The issue has been widely reported in the literature [22, 27, 28].

The primary reason for identification of a small number of alternative splicing biomarkers is the technical differences in sequence coverage and detection sensitivity between RNA-Seq and LC–MS/MS [22].

Converting total RNA into a library of template molecules and making them suitable for high throughput DNA sequencing contains several steps: the poly-A containing mRNA molecules purification, the mRNA fragment, first strand cDNA copy and second strand cDNA synthesis, and PCR purification and enrichment. It becomes possible with these steps to detect reads that span the whole transcript with full coverage and correspond to transcripts at a low-level expression.

On the other hand, the sample preparation involving reduction and alkylation of cysteines, digestion of the sample into peptides, desalting and concentration of the peptides is considerably more critical for accuracy, sensitivity and flexibility of mass spectrometry. A problem is that complex mixture will make mass spectrum difficult to fully analyze due to the overwhelming number of components. This problem is exacerbated when a protein sample is fragmented into a large number of peptide products by enzymatic digestion. Therefore, clean samples and limited sample complexity can minimize the suppression of ionization and prevent MS under-sampling of eluting peptides. As a result of the differences in the RNA and protein measurement, detection of alternative splice peptides is much more difficult at the protein level than at the RNA level.

The second reason for identification of a small number of alternative splice biomarkers is a limitation of proteome plasma samples. One concern is that cancer-specific biomarkers in plasma are probably indicators of a systemic response to cancer or other diseases and may not be secreted directly from cancer tissue [29]. In spite of the concern that using the plasma proteome may reduce overlapping with RNA-Seq breast cancer tissue, we still chose the 80 plasma breast cancer for our experiment platform. One reason is that we couldn’t find any breast cancer tissue proteome datasets with two states (cancer vs normal) in repositories currently available online. Another reason is that human plasma is potentially the single most informative sample that can be collected from an individual with early stage of certain disease [30]. Since blood plasma or serum may contain some residual and potentially detectable combinations of all the differentiated sub-proteomes of the body, plasma may provide information regarding these tissues, and be potentially informative regarding almost any disease state. Blood plasma would appear ideal for detection of cancer biomarker in early stage breast cancer patients [31, 32].

The last reason for identification of a small number of alternative splice biomarkers is Tophat’s splice junction search limitation. Although Tophat was reported to be able to detect short and long range splicing [33], some testing showed that it failed to detect splice junctions that connect distant exons with a satisfied true positive rate, especially for the exons with a distance > 200 kB [34]. On the other hand, the SASD enables users to generate peptide sequences representing all known connections of Ensembl exons/introns as well as junctions representing all possible splicing events for the exons/introns of each gene. Therefore, it is better that the bioinformatics workflow we presented and our SASD searching method work together as complements to each other for identifying alternative splicing biomarkers from proteome. The combination of the alternative splicing biomarkers from the two methods are definitely needed for further experimental validation from other labs or by other methods such as PCR because either the workflow or the SASD is an in-silico method.

The work described here on discovery of alternative splicing biomarkers presents only one important aspect of proteomic variation. For example, there are other variations: Single Nucleotide Polymorphism (SNP), Post-Translational Modification (PTM), Gene Fusion products etc. But the main idea of the workflow: customizing sample-specific RNA-Seq database for proteome searching can work for all the variations. The combination of LC–MS/MS with RNA-Seq will continue to expand the power and utility of this technique for the discovery of all variations as both platforms continue to become increasingly accessible, affordable, popular, and powerful.

## Conclusions

We developed a bioinformatics workflow to discover novel alternative splicing biomarkers from breast cancer proteome using RNA-seq. First, we aligned reads to an annotated reference genome and transcript annotation file, and identified 256 biomarkers from RNA-Seq that align to exons, introns and splice junctions which were differentially expressed in breast cancer. Then, we built a customized sample-specific alternative splicing biomarker peptide database. Lastly, we ran OMSSA against the customized sample-specific peptide database and discovered 26 alternative splicing biomarkers from the proteome. Additional pathway analysis aided in biological understanding of roles associated with these pathways in cancer, including Signaling, Cancer, Regulation, Metabolism, Immune System and Hemostasis.

This workflow integrates the advantages of the two types of platforms: mass spectrometry and next generation sequencing. As a complement to our current SASD method for alternative splicing identification, it can lead to identification of potentially highly sample-specific alternative splicing isoform biomarkers for early detection of cancer.

## Methods and materials

### RNA-Seq for breast cancer data collection

The RNA-Seq data for breast cancer are publicly available through the GEO database with the accession number GSE45419 [35]. It consists of 8 benign breast lesions, 8 ER+, 8 triple negative, and 8 HER2+ primary breast tumors. We downloaded the 32 samples and divided them into two groups: normal group (8 benign breast lesions) and breast cancer group (8 ER+, 8 triple negative, and 8 HER2+ primary breast tumors).

The sequencing files are stored in the NCBI GEO in Sequence Read Archive () format. First, we downloaded the SRA files and converted the SRA files to fastq using the command *bash fastq-dump* <*SRA archive file*> which created a fastq file with the same name as the SRA archive file.

### Human plasma samples

Hoosier Oncology Group (HOG) (Indianapolis, IN, USA) collected the plasma protein data. Totally 80 plasma samples were collected with 40 from women diagnosed with breast cancer and 40 from healthy volunteer woman as controls. The power size for a two-sample *t* test is 0.87 for medium effect and 40 samples each group. Samples were analyzed in a single batch by mass spectrometry. Most cancer patients were diagnosed with a stage II or III or earlier breast cancer. Thermo-Finnigan linear ion trap mass spectrometer (LTQ) was used to analyze Tryptic peptides for protein identification. Peptides were eluted with a linear gradient from 5 to 45% acetonitrile over 120 min. Triple-play mode (MS scan, zoom scan, and MS/MS scan) was used to collect data.

### Bioinformatics workflow for identifying alternative splicing biomarkers from proteome using RNA-Seq

The overall workflow of identifying alternative splicing biomarkers from the proteome using RNA-Seq was comprised of three steps (Fig. 1): (1) identification of alternative splicing biomarkers from RNA-Seq, (2) construction of customized alternative splicing biomarker peptide database, and (3) identification of alternative splicing biomarkers from proteome.

### Step 1: identification of alternative splicing biomarkers from RNA-Seq

#### RNA-Seq splice junction analysis

We first used the Bowtie2 (2.2.1), Tophat (2.0.11), and samtools (0.1.19) to detect all the annotated and unannotated junctions, using default parameters [33, 36–38]. The mate inner distance was set to 150 in Tophat. The alignment of Bowtie–Tophat processing was conducted with a supplied set of Ensembl transcript model annotations in GTF format [39] provided (option-G). Tophat first maped all reads to the transcriptome annotation using Bowtie. All reads that did not map to the transcriptome annotation were set aside as ‘initially unmapped reads’ (IUM reads). The mapped reads were retained and their coordinates are translated to genomic coordinates. Then the remaining reads were broken into sub-fragments of at least 25 bases, and these sub-fragments were aligned to the reference genome. If two adjacent sub-fragments aligned to non-adjacent genomic locations, they were used to infer splice junctions. Finally, TopHat checked whether any of the IUM reads matched any of those putative splice junctions. All splice junctions containing six or more RNA-Seq reads were annotated.

#### Annotation of splice junction

We annotated two categories of alternative splicing junctions. The first category was single exons and single introns, and the second category was the exon/intron junction regions [16]. The first category contained two types of alternative splicing: single exon (EXON_NM) and single intron (INTRON_AS). The second category contained four types of alternative splicing: intron–exon (I_E_AS, left intron retention junction), exon–intron (E_I_AS, right intron retention junction), neighboring exon–exon (E_E_NM, normal splicing junction) and non-neighboring exon–exon (E_E_AS, exon skipping junction).

We extracted the first category of alternative splicing using the “accepted_hits.bam” files produced by the Tophat2. The “accepted_hits.bam” files from Tophat2 were a list of read alignments in SAM format. We extracted the second category of alternative splicing using the “junctions.bed” files reported by Tophat2. The “junctions.bed” files from Tophat2 were a UCSC BED track of junctions with each junction consisting of two connected BED blocks, where each block was as long as the maximal overhang of any read spanning the junction.

**Fig. 7:**
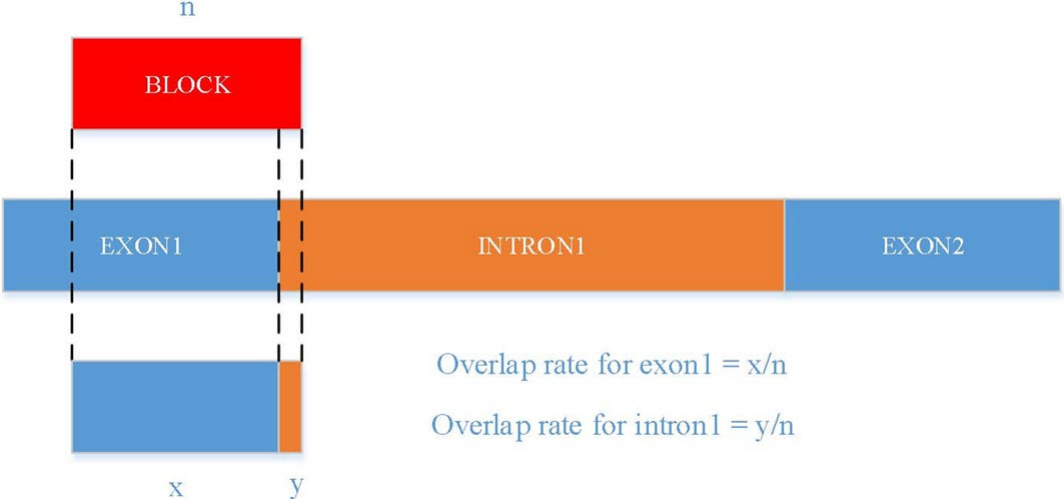
Annotation of each block in junction

We calculated the overlap rates between each block of junctions and each exon or intron within the corresponding transcript (there was one block for EXON_NM and INTRON_AS and there were two blocks for I_E_AS, E_I_AS, E_E_AS, and E_E_NM) using the following equation after extracting the coordinates of each block in junctions from accepted_hits.bam and junctions.bed files (Fig. 7):

$$Overlap\_rate\left( {A, B} \right) = \frac{{\left| {A{\bigcap }B} \right|}}{\left| A \right|},$$

where $\left|A\bigcap B\right|$ is the width of overlap between set A and set B, and $\left|A\right|$ is the width of A.

We assigned the block in junctions to the exon or intron with a minimum overlap rate of 90% or above. The annotation procedure of assigning the optimal indexing number C* is shown in the following equation:

$$C^{*} = \left. {\mathop {{\text{argmax}}}\limits_{C} Overlap\_rate\left( {Region_{C} , T} \right)} \right| Overlap\_rate \ge 90\% ,$$

where *Overlap_rate* is the degree of overlap between each block of junctions and each exon or intron region of the corresponding transcript, *Region* is the exon or intron region in the transcript, *C* is the indexing number of each exon or intron in the transcript, and *T* is the block of junctions.

All data processing was conducted on the Talon2 High Performance Computer at the University of North Texas. The unannotated junctions not matching the Ensembl transcript annotation or not satisfying the requirement of overlap rate greater than or equal to 90% were removed.

#### Identification of alternative splicing biomarkers from RNA-Seq

First, statistical significance was measured by chi-square, which was used to test the null hypothesis that an alternative splicing biomarker was equally represented between normal samples and breast cancer samples. Then we calculated the q value using the Storey–Tibshirani method [40]. We chose a significance screening filter (*q* < 0.05) to select alternative splicing biomarkers, which we used to estimate significant differences between the healthy and breast cancer samples.

### Step 2: construction of customized alternative splicing biomarker peptide database

We used a method similar to the one we developed for SASD [16] to construct a customized alternative splicing biomarker peptide database. All information on the corresponding biomarker genes was extracted from the *Homo sapiens* genes dataset (GRCh37.p13) in the Ensembl Genes 75 database [39, 41], including each biomarker gene’s position, name, exon/intron coordinates, exon phase, sequences, and annotation. We used a relational database hosted in a local SQL server to store and organize all the information.

For each of the second alternative splicing category, 120 nucleotides upstream and downstream of the junction site were extracted, resulting in a computationally synthesized transcript 240 nucleotides long. Similarly, for the first alternative splicing category, 120 nucleotides upstream of the single exon or single intron were extracted to produce a synthesized transcript 120 nucleotides long. A length of 120 nucleotides is determined on basis of the length distribution of fragment from protein digestion in MS/MS experiments.

#### Translation of splicing junction

The translation frame was inferred for splice junctions consisting of exons with known phase information in Ensembl. For all other junctions where either exons didn’t have phase information in Ensembl, or the frame could not be inferred, such as in single intron splicing, all three frames were translated. The translation frame which included the longest peptide was reserved as the AS peptide for mass spectrometry searching.

### Step 3: identification of alternative splicing biomarkers from the proteome

#### Mass spectrometry junction database searching

Raw mass spectrometry files were searched against the customized AS biomarker peptide database using OMSSA [42]. OMSSA is a free program distributed by the NCBI for analyzing and identifying peptides from tandem mass spectrometry peptide spectra. It models the extent of peptide fragmentation and then estimates the probability that an assignment was due to a random match.

OMSSA search results can change with different search parameters, sequence libraries, and samples [43]. Therefore, we created the inverse sequence datasets as a decoy database to calculate the false discovery rate with a target-decoy search strategy [43] and used both the MS/MS false discovery rate (FDR) and E-value as scoring criteria. Peptides passing 1% MS/MS FDR and 0.1 E-value were used for downstream analysis.

### Identification of alternative splicing biomarkers from mass spectrometry

Here, we used the same statistics and thresholds, as described in the “Identification of alternative splicing biomarkers from RNA-Seq” section, for identification of the final AS biomarkers from mass spectrometry. Alternative splicing peptides with significance *q* < 0.05 and at least two hits in either breast cancer samples or normal samples were recognized as AS biomarkers.

### Pathway analysis

Interpretation of biological pathways was conducted with the database we developed: Integrated Pathway Analysis Database (IPAD) (https://fzhang.w3.uvm.edu/ipad/) [44].

## Data Availability

RNA-Seq datasets described in the manuscript can be downloaded from the GEO database (GSE45419). The proteomic data described in the manuscript are available at CPTAC Data Portal (https://cptac-data-portal.georgetown.edu/)*.*

## Abbreviations

NGS: Next-generation sequencing
LC–MS/MS: Liquid chromatography coupled tandem mass spectrometry
IJP: In silico isoform junction peptides
IPAD: Integrated pathway analysis database
SASD: Synthetic alternative splicing database
AS: Alternative splicing
